# Therapeutic Effect of Lebanese Cannabis Oil Extract in the Management of Sodium Orthovanadate-Induced Nephrotoxicity in Rats

**DOI:** 10.3390/ijms26094142

**Published:** 2025-04-27

**Authors:** Christabel Habchy, Alia Khalil, Wassim Shebaby, Diana Bylan, Marissa El Hage, Mona Saad, Selim Nasser, Wissam H. Faour, Mohamad Mroueh

**Affiliations:** 1 Pharmaceutical Sciences Department, School of Pharmacy, Lebanese American University, Byblos P.O. Box 36, Lebanon; christabel.habchy@lau.edu (C.H.); wassim.shebaby@lau.edu.lb (W.S.);; 2 Gilbert and Rose-Marie Chagoury School of Medicine Room 4722, Lebanese American University, Byblos P.O. Box 36, Lebanonsaadmona013@outlook.com (M.S.); selim.nasser@lau.edu.lb (S.N.)

**Keywords:** sodium orthovanadate, cannabis oil extract, nephrotoxicity, podocytes, AKT, p38, phosphatase inhibitors

## Abstract

Sodium orthovanadate is a non-selective protein tyrosine phosphatase inhibitor that can cause several types of kidney injury, including glomerulosclerosis, inflammation, and tubular damage. Cannabis is widely known for its medicinal use, and several studies have demonstrated its anti-diabetic and anti-inflammatory properties. The current study investigated the therapeutic effect of Lebanese cannabis oil extract (COE) against sodium orthovanadate-induced nephrotoxicity both in vitro and in vivo. Sprague Dawley male rats were intraperitoneally injected with 10 mg/kg sodium orthovanadate for 10 days followed by 5 mg/kg; 10 mg/kg; or 20 mg/kg intraperitoneal injection of cannabis oil extract, starting on day 4 until day 10. The body weight of the rats was monitored during the study, and clinical parameters, including serum urea, creatinine, and electrolytes, as well as kidney and heart pathology, were measured. Conditionally immortalized cultured rat podocytes were exposed to either sodium orthovanadate or selective phosphatase inhibitors, including DUSPi (DUSP1/6 inhibitor) and SF1670 (PTEN inhibitor), in the presence or absence of cannabis oil extract. MTS and an in vitro scratch assay were used to assess podocyte cell viability and migration, respectively. Western blot analysis was used to evaluate the phosphorylation levels of AKT and p38 MAPK. Rats injected with sodium orthovanadate displayed a marked reduction in body weight and an increase in serum creatinine and urea in comparison to the control non-treated group. All doses of COE caused a significant decrease in serum urea, with a significant decrease in serum creatinine observed at a dose of 20 mg/kg. Moreover, the COE treatment of rats injected with orthovanadate (20 mg/kg) showed a marked reduction in renal vascular dilatation, scattered foci of acute tubular necrosis, and numerous mitoses in tubular cells compared to the sodium orthovanadate-treated group. The cell viability assay revealed that COE reversed cytotoxicity induced by sodium orthovanadate and specific phosphatase inhibitors (DUSPi and SF1670) in rat podocytes. The in vitro scratch assay showed that COE partially restored the migratory capacity of podocytes incubated with DUSPi and SF1670. Time-course and dose-dependent experiments showed that COE (1 μg/mL) induced a significant increase in phospho-(S473)-AKT, along with a decrease in phospho (T180 + Y182) P38 levels. The current results demonstrated that Lebanese cannabis oil possesses important kidney protective effects against sodium orthovanadate-induced renal injury.

## 1. Introduction

Kidney diseases are a worldwide public health problem, especially in developed countries, where more than 10% of adults have CKD [1]. Lebanon has one of the highest rates of dialysis prevalence in the world, with an estimated 777 patients per million people compared to 410 patients per million people worldwide [2]. Acute kidney injury (AKI) is defined as a sudden and often reversible decrease in kidney function. It is characterized by a sudden and significant drop in glomerular filtration rate, increased serum creatinine, and/or decreased urine volume output. Intrinsic renal causes include conditions that affect the glomerulus or tubules, such as acute tubular necrosis (ATN), acute interstitial nephritis (AIN), and glomerulonephritis (GN) [3].

Vanadium is a group 5d transition metal. It is the 18th most abundant element in the Earth’s crust and is found in soil, water, air, and living organisms. It is listed as one of the 40 essential micronutrients and is required in trace amounts for the normal metabolism, growth, and development of mammals. Vanadium-rich foods include mushrooms, dill seed, parsley, and black pepper, as well as cereals, fresh fruits, and shellfish. Because of the combustion of carbon-based fossil fuels, vanadium compounds are also present in air, soil, and water reservoir contaminants in large urban agglomerations [4].

Vanadium can form a variety of inorganic compounds, including sodium orthovanadate (Na_3_VO_4_), also known as vanadate, which is a phosphate analog that non-selectively inhibits protein tyrosine phosphatases (PTPs), alkaline phosphatases, and ATPases. In addition, it can slow the growth of tumors in the central nervous system, lung cancer, prostate cancer, bladder cancer, and liver cancer [5].

Vanadate is primarily cleared from the body by the kidneys. Vanadate can effectively accumulate in kidney proximal tubule cells [6], causing tubular cellular cytotoxicity [7,8]. PTEN is a dual-function tumor suppressor protein and phospholipid phosphatase that regulates a large number of vital cellular processes, including cell growth, adhesion, and migration [9]. The primary substrate of PTEN is phosphatidylinositol (3,4,5)-trisphosphate (PIP3) PIP3, which is an upstream lipid second messenger of the AKT/PKB kinase pathway [10]. PTEN can dephosphorylate and inhibit the activity of AKT. Of note, AKT is a master survival pathway in kidney podocytes, and the inhibition of AKT activity induces podocyte apoptosis [11].

Recent studies have indicated that PTEN is involved in kidney development and the pathogenesis of renal diseases. Accordingly, PTEN loss or deletion is associated with renal fibrosis [12], proximal tubule cell hypertrophy [13], and aggravated diabetic nephropathy [14].

Dual-specificity phosphatases (DUSPs) belong to a specialized family of phosphates, which include 25 phosphatases known to dephosphorylate both serine/threonine and tyrosine residues. They regulate several important biological and pathological processes, including cell proliferation, migration, differentiation, inflammation, and apoptosis [15].

Mitogen-activated protein kinases (MAPKs), including ERK1/2, JNK1/2, and p38, are the main substrates for DUSP; therefore, DUSP is also referred to as an MAPK phosphatase (MKP) [16]. Previous studies have demonstrated the deleterious effect of P38 MAPK on podocyte survival. Accordingly, the profibrotic factor TGF-beta, which induces podocyte cell death, is mediated through the induction of P38 MAPK activity [17]. Finally, the damage or loss of podocytes can cause glomerulosclerosis [18].

Several studies have suggested the involvement of DUSPs in the pathogenesis of various diseases, such as neurological disorders [19] and kidney diseases. Accordingly, decreased DUSP expression has been reported in several kidney disease models, including hypertensive nephropathy, diabetic nephropathy, acute kidney injury, chronic kidney diseases, and lupus nephritis [20]. We previously showed that DUSP inhibitors induced P38 MAPK activation and COX-2 protein translation in cultured immortalized rat podocytes incubated with either TGF-beta1 or Prostaglandin E2 [21].

Cannabis belongs to three plants species: *Cannabis sativa*, *Cannabis indica*, and *Cannabis ruderalis*. It is an aromatic annual herb that has been widely grown throughout history and harvested for its oil, seeds, and fiber [22]. Cannabis has well-established anti-inflammatory, anti-emetic, anti-epileptic, and diuretic properties [23]. Cannabis oil was traditionally used to treat a variety of diseases, including diabetes, chronic pain conditions such as arthritis, and cancer [24]. We previously described the detailed chemical constituents of Lebanese cannabis and showed that Lebanese cannabis extract showed strong anti-inflammatory [25] and anti-cancer effects, and protected the kidney against folic acid-induced kidney fibrosis and cisplatin-induced nephrotoxicity [26,27].

Cannabinoids, terpenes, and phenolic compounds are the most common phytochemicals found in cannabis plants. Cannabidiol (CBD) and tetrahydrocannabinol (THC) are the two main components of cannabis oil extract [28]. CBD, a non-psychoactive cannabinoid, has numerous pharmacological properties, including antioxidant, anti-inflammatory, antimicrobial, anxiolytic, and anti-cancer properties [29,30]. THC, the psychoactive cannabinoid compound, is primarily used to treat pain, cancer, multiple sclerosis, and neurodegenerative disorders [31].

The potential role of the endocannabinoid system in treating nephrotoxicity is a promising area of research. Accordingly, selective CB_1_ and CB_2_ receptor agonists showed a dose-dependent protective effect in a renal ischemia/reperfusion injury mouse model [32]. Moreover, cannabidiol decreased renal tubular injury in rats following bilateral renal ischemia/reperfusion by preventing increases in serum creatinine, nitric oxide, and renal malondialdehyde levels [33]. Therefore, we believe that cannabis oil has a protective effect against vanadium-induced nephrotoxicity.

## 2. Results

### 2.1. DUSPi, SF1670, and Sodium Orthovanadate Reduced Podocyte Cell Viability in a Dose-Dependent Matter

**Figure 1:**
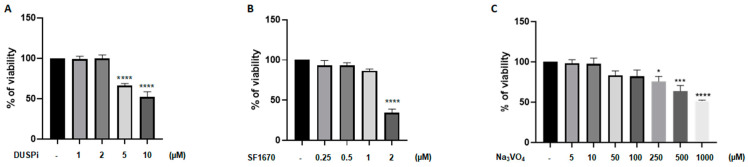
**Effect of DUSPi, SF1670, and Na_3_VO_4_ on podocyte cell viability**. Rat podocytes were treated with indicated concentrations of DUSPi (**A**), SF1670 (**B**), and Na_3_VO_4_ (**C**) for 24 h. Cell viability was evaluated using Cell Titer 96 Aqueous Non-Radioactive Cell Proliferation Assay Kit. Data are expressed as mean ± SEM. Differences between groups were evaluated using one-way ANOVA followed by Bonferroni’s multiple comparison test. * *p* < 0.05; *** *p* < 0.001; **** *p* < 0.0001—significantly different from control.

We first evaluated the effect of phosphatase inhibitors on podocyte cell viability. As shown in Figure 1, podocyte cells were treated with a range of DUSPi, SF1670, and sodium orthovanadate (Na_3_VO_4_) concentrations, and their cytotoxic effect was investigated using an MTS cell proliferation assay. The results showed a dose-dependent cytotoxic effect of the three inhibitors with an IC50 of 10 μM, 2 μM, and 1 mM for BCI, SF1670, and Na_3_VO_4_ after 24 h of incubation, respectively (Figure 1A–C).

### 2.2. COE Significantly Reversed the Cytotoxic Effect of Phosphatase Inhibitors on Cultured Podocytes

**Figure 2:**
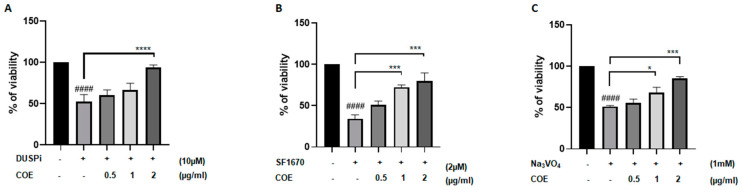
**Cannabis oil extract protects podocyte cells from phosphatase inhibitor-induced cytotoxicity**. Immortalized rat podocytes were treated with (**A**) 10 μM DUSPi, (**B**) 2 μM SF1670, and (**C**) 1 mM Na_3_VO_4_ in the absence or presence of cannabis oil extract (COE) at the indicated concentrations for 24 h. Cell viability was evaluated via Cell Titer 96 Aqueous Non-Radioactive Cell Proliferation Assay. Data are expressed as mean ± SEM (*n* = 3). Differences between groups were evaluated using one-way ANOVA followed by Bonferroni’s multiple comparison test. #### *p* < 0.0001 vs. control; * *p* < 0.05; *** *p* < 0.001; **** *p* < 0.0001—significantly different from the DUSPi/SF1670 or Na_3_VO_4_-only treated group.

To test whether COE protects podocyte cells against the cytotoxic effect of phosphatase inhibitors, cells were treated with 10 μM DUSPi, 2 μM SF1670, and 1 mM Na_3_VO_4_ in the presence or absence of 0.5, 1, and 2 μg/mL COE for 24 h, respectively (Figure 2A–C). Interestingly, the cell viability assay showed that COE attenuated the cytotoxic effect of all three phosphatase inhibitors in a concentration-dependent manner in podocyte cells (Figure 2).

### 2.3. COE Partially Reversed Migration Inhibition in Podocytes Treated with Phosphatase Inhibitors

**Figure 3:**
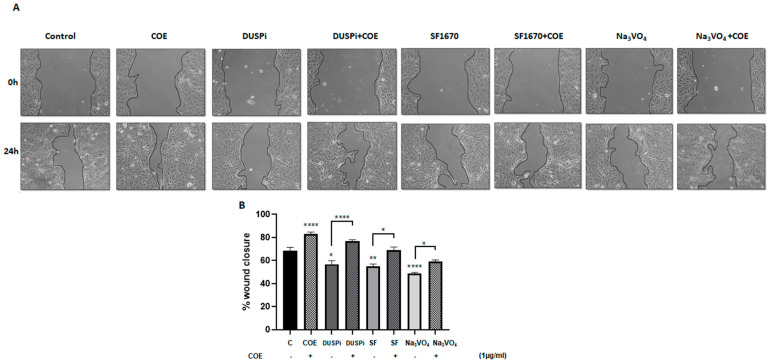
**Podocyte cell migration.** (**A**) Representative pictures show the migration of podocyte cells after induction of a scratch representing a wound. All the pictures were taken immediately after the scratch was induced (at zero hours) and 24 h after incision. Podocytes in the pictures were cultured in different conditions, as indicated. Pictures are taken at 10 times magnification. (**B**) The relative change in cell-free gap surfaces was measured at different time points after the creation of the wounding scratch and is expressed as fold change over zero time. Results are expressed as mean ± SEM of three independent experiments performed in triplicate. Statistical analysis was performed using one-way ANOVA followed by Bonferroni’s multiple comparison test. * *p* < 0.05; ** *p* < 0.01; **** *p* < 0.0001. Scale bar, 100 µM (condition Na_3_VO_4_/24 h).

An in vitro scratch assay was performed in order to measure the rate of podocyte cell migration in the presence of 2 μM DUSPi, 1 μM SF1670, and 10 μM Na_3_VO_4_, in the presence or absence of 1 μg/mL COE. As illustrated in Figure 3, COE accelerated wound closure (82 ± 5.47%) when compared to the control non-treated cells (68 ± 7.9%). DUSPi, SF1670, and Na_3_VO_4_ significantly slowed down the closure of the cell-free gap after 24 h by 56.7 ± 15%, 55.2 ± 3.6%, and 48.6 ± 2.6%, respectively. The addition of COE partially reversed the inhibitory effect of DUSPi, SF1670, and Na_3_VO_4_, and restored wound closure by 77 ± 2.8%, 69 ± 4.8%, and 59.2 ± 3.3%, respectively.

### 2.4. COE Regulated AKT and P38 MAPK Phosphorylation Levels and Protected Podocytes from Orthovanadate by Inhibiting Apoptosis and Oxidative Stress

**Figure 4:**
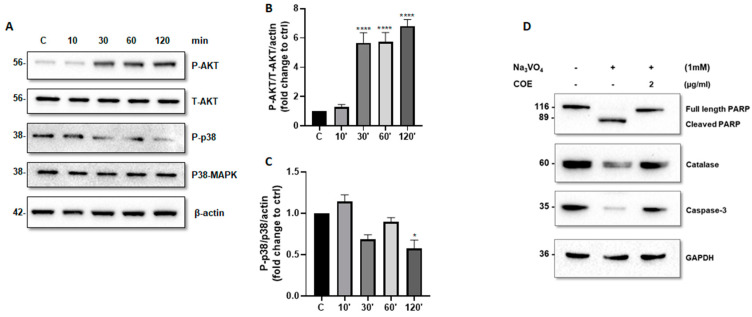
Cannabis oil extract protects podocyte cells from sodium orthovanadate by inhibiting apoptosis and modulating AKT and p38 protein phosphorylation. (**A**) Cultured immortalized rat podocytes were treated with COE (1 μg/mL) for different durations of time (10–120 min), as indicated. Cell extracts (50 μg) were analyzed by Western blotting using anti-phospho-AKT1 (S473), anti-phospho-p38 (phospho T180 + Y182), Anti-AKT1 + AKT2 + AKT3 antibody, and anti p38 alpha/beta MAPK antibodies. The levels of phospho-AKT-(S473) and phospho-p38 were normalized to total AKT (**B**) and p38 MAPK (**C**), respectively, then to actin protein content, and the signal intensity was identified by densitometry. Data are expressed as mean ± SD (*n* = 3). Differences between groups were evaluated using one-way ANOVA followed by Bonferroni’s multiple comparison test. * *p* < 0.01; **** *p* < 0.0001 vs. control. (**D**) Cultured immortalized rat podocytes were treated with sodium orthovanadate Na_3_VO_4_ (1 mM) for 24 h in the presence or absence of COE (2 μg/mL). Cell extracts (50 μg) were analyzed by Western blotting using anti-PARP, anti-caspase-3, and anti-catalase antibodies. GAPDH was used as a loading control.

We further investigated whether COE can modulate the activity of AKT and P38 MAPK. Accordingly, time-course experiments showed that COE (1 μg/mL) induced a significant increase in phospho-(S473)-AKT levels (Figure 4A,B) and a significant decrease in phospho-(T180 + Y182) P38 MAPK levels (Figure 4A,C).

Furthermore, COE treatment protected podocytes from orthovanadate-induced apoptosis and oxidative stress by reducing the cleavage of caspase-3 (Figure 4D) and PARP (Figure 4D), on one hand, and by restoring the expression of the antioxidant enzyme catalase on the other hand, as compared to orthovanadate-only treated cells.

### 2.5. Evaluation of Body, Absolute, and Relative Organ Weights

As shown in Table 1, orthovanadate treatment resulted in a significant loss of body weight in both the orthovanadate- and orthovanadate + COE (5, 10, and 20 mg/kg)-treated groups, as compared to the control. No significant change in absolute kidney weight was observed in any of the treated groups; however, a significant increase in relative kidney weight was observed in the orthovanadate + COE 10 mg/kg (0.47 ± 0.01 g)-treated rats compared to the control rats (0.42 ± 0.01 g). Finally, significant decrease in absolute heart weight was observed in both the orthovanadate + COE 5 mg/kg (0.58 ± 0.03 g)-treated and orthovanadate + COE 10 mg/kg (0.59 ± 0.04 g)-treated rats compared to the control rat group (0.74 ± 0.02 g). No significant change in relative heart weight was observed among the different groups.

### 2.6. Evaluation of Renal Biomarkers

**Figure 5:**
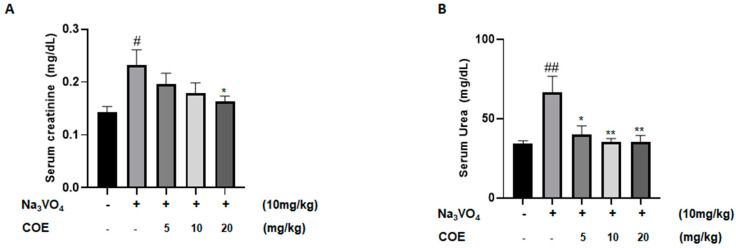
**Average serum creatinine levels (mg/dL) (A); average serum urea levels (mg/dL) (B) in different groups of rats.** Rats were intraperitoneally injected with 10 mg/kg sodium orthovanadate daily for 10 days. COE was injected daily from day 4 to day 10. Serum urea and creatinine levels were measured. Each column represents the mean ± SEM of seven animals. Statistical analysis was performed using two-way ANOVA followed by Bonferroni’s multiple comparison test. # *p* < 0.05; ## *p* < 0.01 vs. control; * *p* < 0.05; ** *p* < 0.01—significantly different from the sodium orthovanadate-only treated group.

Levels of serum creatinine were measured in order to investigate the effect of cannabis oil extract on sodium orthovanadate-induced renal toxicity. The survival rate at the assigned sacrifice day of the experiment was 100%. As seen in Figure 5A, the administration of sodium orthovanadate at a dose of 10 mg/kg daily for 10 days led to a significant increase in serum creatinine levels compared to the control group. In addition, treatment with cannabis oil extract at a dose of 20 mg/kg for 7 days led to a significant decrease in serum creatinine levels compared to the sodium orthovanadate-treated group.

Levels of serum urea were measured to investigate the effect of cannabis oil extract on sodium orthovanadate-induced renal toxicity. As seen in Figure 5B, the administration of sodium orthovanadate at a dose of 10 mg/kg daily for 10 days led to a significant increase in serum urea levels compared to the control group. Treatment with cannabis oil extract at doses of 5 mg/kg, 10 mg/kg, and 20 mg/kg for 7 days led to a significant decrease in serum urea levels compared to the sodium orthovanadate-treated group.

There were no significant differences in the average serum sodium, potassium, or chloride levels between any of the groups (Table 2).

### 2.7. Histopathological Findings of Renal Tissue

**Figure 6:**
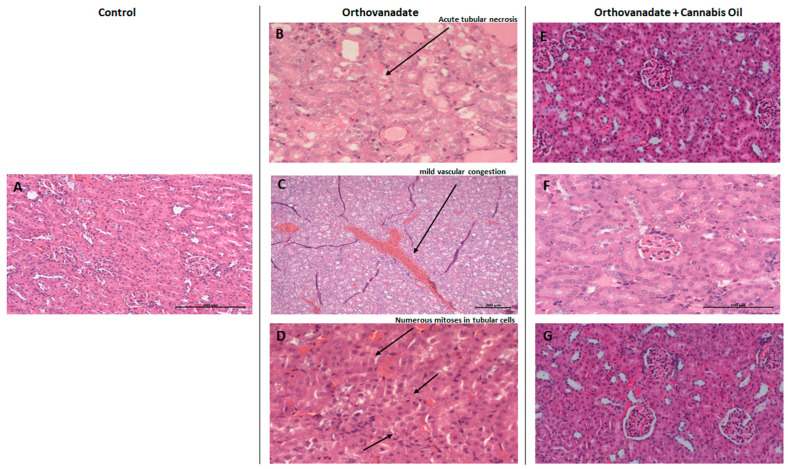
**Micrograph of renal sections of rats from different groups (H&E stained).** (**A**) Control rats showing renal parenchyma with no specific changes (enlargement: ×200); (**B**–**D**) sodium orthovanadate-treated rats demonstrating (**B**) acute tubular necrosis (enlargement: ×400), (**C**) renal parenchyma with mild vascular congestion (enlargement: ×100) (scale bar 200 µM: (**C**)), and (**D**) numerous mitoses in tubular cells (enlargement: ×400). Cannabis-treated rats at different doses—(**E**) 5 mg/kg (enlargement: ×200), (**F**) 10 mg/kg (enlargement: ×400), and (**G**) 20 mg/kg (enlargement: ×200)—showing renal parenchyma with no specific changes (scale bar 100 µM: (**F**)).

In the control group, Hematoxylin and Eosin staining of kidney sections showed no specific pathological deterioration or changes in the glomeruli, tubules, interstitial tissues, and/or peritubular capillaries. It was evident that the injection of sodium orthovanadate at a dose of 10 mg/kg daily for 10 days evoked marked pathological abnormalities in 40% of the treated rats compared with the control group treated with the vehicle solution. Microscopic renal examination of the sodium orthovanadate-treated rats revealed acute tubular injuries, including vascular dilatation and scattered foci secondary to acute tubular necrosis (Figure 6B,C). In addition, numerous mitosis in tubular cells were also observed (Figure 6D). Rats that were administered with sodium orthovanadate and cannabis oil at doses of 5 mg/kg, 10 mg/kg, and 20 mg/kg showed normal glomerular and tubular structures, with an absence of inflammation, cysts, glomerular collapse, and crystal deposition (Figure 6E–G). There were no significant findings (inflammation, deposition, or myocyte injury) in the heart tissues of any rats.

## 3. Discussion

Sodium orthovanadate (Na_3_VO_4_) is a well-known general protein phosphatase inhibitor. However, the systemic toxicity of vanadium-based compounds limits their use as potential therapeutic agents [5].

In fact, vanadium toxicity is associated with the increased production of reactive oxygen species (ROS) and alterations in enzymatic antioxidant defense pathways, mainly in kidney tissues. Furthermore, vanadium accumulation might cause distal renal tubular acidosis (dRTA), “uremic syndrome”, renal stone disease, or acquired cystic kidney disease [34]. Several studies have investigated the antioxidant effect of plant-derived formulations against vanadium-induced nephrotoxicity [8]. However, very limited studies have attempted to evaluate the protective effect of cannabis against kidney injury. The data remain inconclusive, but none of these studies evaluated the effect of cannabis in an in vivo vanadate-induced nephrotoxicity model.

In our model, cannabis oil was able to reverse the nephrotoxic effect of vanadate. The protective effect of cannabis is highlighted by its ability to significantly decrease serum creatinine and blood urea nitrogen (BUN) levels. Podocytes are major components of the functional glomerular filtration barrier. Thus, we wanted to further evaluate the effect of cannabis on podocytes exposed to vanadate, as well as on the key signaling pathways involved in podocyte cell death and survival, including AKT and P38 MAPK. Of note, AKT is a podocyte pro-survival pathway, and its inhibition by various stressors, e.g., TGF-beta, can cause podocyte apoptosis. Furthermore, P38 MAPK is a well-known proapoptotic pathway in kidney podocytes, and its activation has deleterious effects on podocyte health [18,35]. Interestingly, the activities of these kinases can also be specifically modulated by PTEN and DUSP (dual-specificity phosphatase), respectively. Accordingly, SF1670 and DUSP1/6 inhibitors were chosen in addition to sodium orthovanadate as general phosphatase inhibitors.

Our results corroborated previous findings in podocytes. Accordingly, cell viability was reduced by 50% upon treatment with 10 µM, 2 µM, and 1 mM of DUSP1/6i, SF1670, and sodium orthovanadate, respectively. These results are also in agreement with several studies in which DUSP1/6i reduced cell viability in other cell lines, including Raw 264.7 murine macrophages, BMM—bone marrow monocytes/macrophages [36]—and different neuroblastoma cell lines (SK-N-AS, KELLY, IMR-32, and LAN-1) [37]. Similarly, vanadium compounds were shown to reduce cell viability against normal HEK-293 human embryonic kidney cells [38], as well as cancer cell lines [5]. Surprisingly, treatment with the PTEN inhibitor (SF1670) impaired cell viability. The same observations were reported by Minaei Beyrami et al. (2018); in their study, SF1670 reduced the cell viability of pheochromocytoma-derived cell line PC12 [39]. This paradoxical effect of SF1670 could be explained by the dual roles of PTEN in the cells. Accordingly, Zhou et al. (2017) reported that the inhibition of PTEN with Bisperoxovanadium (HOpic) bpV (HOpic), a potent PTEN inhibitor, enhanced apoptotic cell death in the kidneys with ischemia/reperfusion injury (IRI) [40]. In keeping with COE, an in vitro analysis showed that COE protected podocyte cells against the cytotoxic effect of phosphatase inhibitors in a concentration-dependent manner. In general, the biologic effect of COE depends upon its oil chemical constituents, which can largely vary according to the country of origin and the geographic location from which it was harvested. Consistently, CBD has been shown to exert protective effects against hydrogen peroxide in hippocampal neuron culture [41] and against high-glucose-induced arrhythmia and cytotoxicity [42], and also to alleviate UVB-induced cytotoxicity in human keratinocyte HaCaT cells [43].

In addition to the decrease in cell viability, all three phosphatase inhibitors reduced cell migration, which was partially reversed by COE. Of note, the concentrations used in the wound healing assay for the three inhibitors were not toxic to podocytes. Thus, the latter effect is not related to the reduction in cell viability.

Other studies have reported conflicting data on cell migration using BCI or DUSP1/6i and/or its downstream targets (DUSP1 and 6) depending on several factors, including cell type, concentration, and mechanism of action, among others. For example, DUPS1 was reported to inhibit cell migration during RSV infection in A549 lung cancer cells [44], while on the contrary, it promoted invasion and metastasis in non-small cell lung cancer [45].

Similarly, DUSP6 impaired cell migration in esophageal squamous cell and nasopharyngeal carcinoma, but oppositely, it increased cell migration in an MDA-MB-231 breast cancer cell line and gastric cancer. The pharmacological inhibition of DUSP6 with BCI suppressed tumor migration and invasion [46,47].

Vanadium compounds, including sodium orthovanadate, are mostly evaluated in cancer research and have been shown to reduce cell migration and metastasis [5].

Regarding SF1670 and its downstream target PTEN, and subsequently the pro-survival and pro-migratory AKT pathway, SF1670 treatment is associated with increased cell migration [48,49].

In keeping with the kidney, published data on podocytes show that the deletion of another member of the DUSP family, “DUSP4”, is associated with profound podocyte foot process effacement, cell death, sustained P38 activation, and exacerbated albuminuria and glomerular fibrosis in diabetic mice [50]. While our data corroborate the finding that the DUSP inhibitor induces podocyte cell death, a novel finding is that COE reverses the inhibitor’s toxic effect.

Of note, podocyte foot process effacement is reminiscent of the denudated zone created in in vitro scratch assays. Moreover, similar structural and morphological changes, including reorganization of the actin cytoskeleton and focal contacts, accompanied by the retraction and detachment of podocytes from the extracellular matrix (ECM), were reported in a model of podocytes treated with 40 µM sodium vanadate [51].

Finally, in a murine model of the podocyte-specific inducible deletion of PTEN, moderate foot process effacement was observed and was associated with actin cytoskeletal rearrangement and increased albumin excretion [14].

Considering the effect of COE on cell migration alone or in the presence of different phosphatase inhibitors, in vitro wound closure is also governed by the same aforementioned factors. Accordingly, the cannabis-derived compounds cannabichromene and D9-Tetrahydrocannabinol and a CBD-THC combination inhibited urothelial cell carcinoma and multiple myeloma cell migration, respectively [52,53]. On the contrary, cannabidiol significantly improved wound healing in Human Brain Endothelial Cells and fibroblasts [54,55].

Mechanistically, we further studied the effect of COE on AKT and P38 MAPK in order to elucidate the potential molecular mechanisms by which COE exerts its protective effect. Accordingly, time-course and dose-dependent experiments showed that COE significantly increased phospho-(S473)-AKT levels, while it significantly reduced phospho (T180 + Y182) P38 MAPK levels. Thus, this highlights that the potential nephroprotective role of COE is in part mediated through an increase in podocyte survival. Of note, it was found that the protective effect of CBD in an experimental multiple sclerosis model was mediated by increased phospho-AKT levels and reduced P38 MAPK activity [56]. On the contrary, in human glioblastoma multiform (GBM), the pro-apoptotic activities of CBD were associated with enhanced JNK1/2 and P38 MAPK signaling cascades, on one hand, but also through the inhibition of the pro-survival PI3K/AKT signaling cascade [57].

Moreover, our in vitro results demonstrated that COE protected podocytes from orthovanadate-induced oxidative stress and apoptosis by restoring the expression of the antioxidant enzyme catalase and reducing the cleavage of caspase-3 and PARP, respectively. Of note, the inhibition of apoptosis by Lebanese COE was previously reported by our team in a model of cisplatin-induced podocyte cytotoxicity [27].

In our kidney model, the administration of sodium orthovanadate at a dose of 10 mg/kg daily for 10 days resulted in a significant decrease in body weight gain in comparison to the control group (58.38 ± 5.32 versus 85.50 ± 1.73). These data are in agreement with previous studies reporting significant decreases in body weight following vanadate administration in rats [58,59]. In addition, there was a significant increase in rats’ relative kidney weight after administering 10 mg/kg of sodium orthovanadate daily for 10 days compared to the control rats (0.45 ± 0.02 versus 0.42 ± 0.01). A previous study revealed an increase in rat kidney weight after the administration of the vanadium compound [59].

Moreover, the absolute heart weight of rats taking sodium orthovanadate 10 mg/kg daily for 10 days was significantly lower than the control group rats (0.64 ± 0.03 vs. 0.74 ± 0.02). As a result, pre-renal nephrotoxicity will cause a decrease in the glomerular filtration rate (GFR).

A rise in serum creatinine levels in rats treated with sodium orthovanadate was observed compared with the control (0.23 ± 0.08 vs. 0.14 ± 0.03), indicating renal injury. A decline in serum creatinine levels was detected in rats treated with both sodium orthovanadate and cannabis oil extract at a dose of 20 mg/kg compared to rats taking sodium orthovanadate alone (0.16 ± 0.03 vs. 0.23 ± 0.08). Moreover, the administration of sodium orthovanadate led to an increase in serum urea levels compared with the control (66.50 ± 28.83 vs. 34.38 ± 4.68), implying nephrotoxicity.

Increased serum creatinine and urea levels in vanadium-treated rats was also documented in a previous study [60]. Interestingly, our data confirmed the previously published data on vanadate. However, we showed that COE was able to reverse the toxic effect of vanadate on kidney function, which went back to normal following COE treatment.

The decrease in serum urea levels in rats treated with sodium orthovanadate and cannabis oil extract represents a promising finding. In fact, the nephroprotective effect of cannabidiol at doses of 5 mg/kg and 10 mg/kg revealed a decrease in serum creatinine and urea levels. Our findings showed that the administration of higher doses of cannabis oil extract at 20 mg/kg could also play an important role in kidney function repair, highlighting a promising approach to protect against sodium orthovanadate-induced nephrotoxicity.

There were no observable changes in serum electrolyte levels between the different groups, which is in agreement with previous in vivo experiments demonstrating renal toxicities induced by the vanadium compound [61]. The lack of effect on serum electrolyte levels can be explained by the reversible moderate damage to the kidneys, which allowed renal compensation.

Previous studies have reported that vanadium tends to accumulate in the kidney, predisposing patients to nephrotoxicity [62,63]. The administration of sodium orthovanadate at a dose of 10 mg/kg daily for 10 days evoked marked major signs of renal nephrotoxicity, including vascular dilatation, scattered foci of acute tubular necrosis, and numerous mitoses in tubular cells. Vanadate caused observable pathological damage. A remarkable reduction in pathology lesions was observed with COE treatment, which was compatible with the normal levels of renal function biomarkers, including serum creatinine and urea.

The mechanisms by which cannabinoid receptors induce tubular cell survival following acute damage are not well defined. However, the possible underlying mechanisms in improving kidney structure and function might include an antagonism of the CB_1_ receptor and/or activation of the CB_2_ receptor. THC acts a partial agonist at the CB_1_ and CB_2_ receptors, whereas CBD acts as CB_1_ antagonist [64] and as a negative allosteric modulator at the CB_2_ receptor [65]. The blockade of the CB_1_ receptor and/or activation of the CB_2_ receptor was shown to be protective against tubular damage by attenuating renal oxidative stress and inflammation [66]. Jourdan et al. (2014) previously showed that the overactivation of CB1 in podocytes can lead to diabetic nephropathy (DN) [67]. Furthermore, the deletion of CB1 in podocytes prevented glomerular and tubular dysfunction in an STZ-induced type 1 diabetes animal model [68]. Finally, Barutta et al. (2018) showed that a peripherally acting CB1 antagonist can be used as an add-on treatment to ACE inhibitors in DN [69].

A previous study revealed that possible pathways underlying cannabis activity include activation of the CB_2_ receptor, causing a decreased infiltration of immune cells, specifically leukocytes, into kidney tissues, and thus attenuating inflammatory cytokine release [70]. Moreover, in animal models of acute kidney injury, CB_1_ receptor activation is associated with an increased production of reactive oxygen species, which can activate the transcription of downstream proinflammatory target genes, leading to the activation of apoptosis [58]. Hence, cannabis oil administration may have played a role in inhibiting CB_1_ receptor activation and blocking the production of inflammatory proteins, therefore decreasing cell injury and death. In summary, our results suggest that Lebanese cannabis oil extract may be of significant therapeutic benefit against the nephrotoxic effects of sodium orthovanadate, in part by modulating the key signaling kinases involved in podocyte survival.

## 4. Materials and Methods

### 4.1. Plant Collection and Oil Extraction

Dried samples of the Lebanese cannabis strain were provided through the Drug Enforcement Office. Plant extract was prepared as previously described [25]. Briefly, a sample of 10 g of air-dried cannabis flower was extracted with ethanol for 48 h. The extract was filtered and concentrated at 45 °C under reduced pressure to yield 1.17 g of cannabis oil extract (COE).

### 4.2. Cell Culture

Conditionally immortalized rat glomerular epithelial cells (podocytes), kindly provided by Dr. Assaad Eid (American University of Beirut), were cultured as previously published [71]. Briefly, podocytes cells were maintained in RPMI media supplemented with 10% Fetal Bovine Serum (FBS) and Penicillin–Streptomycin (100 U/mL penicillin, 100 μg/mL streptomycin) at 37 °C in a humidified incubator under a pressure of 5% CO_2_.

### 4.3. Cell Viability Assay

Podocytes cells were seeded in 96-well plates (8000 cells/well). The following day, the cells were incubated in serum-free medium composed of 0.1% FBS. After overnight starvation, cells were treated with increasing concentrations of dual-specificity protein phosphatase1/6 inhibitor, BCI or DUSPi (calbiochem), SF1670 (PTPN2 and PTEN inhibitor, TOCRIS, Minneapolis, MN, USA), or sodium orthovanadate (Na_3_VO_4_, sigma, St. Louis, MO, USA) for 24 h. In other experiments, podocytes cells were treated with 10 μM DUSPi, 2 μM SF1670, or 1 mM Na_3_VO_4_ in the presence or absence of COE (0.5, 1, and 2 µg/mL) for 24 h. The viability of the cells was assessed using the Cell Titer 96 AQueous Non-Radioactive Cell Proliferation Assay Kit (Promega, Madison, WI, USA), which is a colorimetric method based on the reaction of mitochondrial dehydrogenase with 3-(4,5-dimethylthiazol-2-yl)-5-(3-carboxylmethoxyphenyl)-2-(4-sulfophenyl)-2H-tetrazolium inner salt (MTS) as the reagent. Briefly, 20 µL of reagent solution was added to each well of the 96-well plate. After incubation at 37 °C in humidified 5% CO_2_ for 1 h, absorbance was read at 490 nm using an Elisa microplate reader. The percentage of cell proliferation was determined using the following formula:

$$\frac{\mathrm{Absorbance}\;\mathrm{test}\;\mathrm{well}}{\mathrm{Absorbance}\;\mathrm{control}\;\mathrm{well}}\times 100$$

### 4.4. Wound Healing Assay

Podocytes cells were counted using a hemocytometer and plated in 12-well plates at a density of 1 × 10^4^ cells per well. The plates were incubated overnight under growth conditions to allow for cell recovery and exponential growth. After overnight incubation, the cells were serum-starved in 0.1% FBS RPMI media for 24 h.

A mechanical scratch representing a wound was created in the near-confluent monolayer of cells by gently scraping with a sterile 200 μL micropipette tip. The cells were then rinsed with serum-free RPMI and treated with 2 μM DUSPi, 1 μM SF1670, and 10 μM Na_3_VO_4_ in the presence or absence of 1 µg/mL COE. Scratch width was photographed at two time points (0 and 24 h) from three fields of view with the 10× objective using light microscopy. The experiments were performed in triplicate. Using ImageJ software (https://imagej.net/ij/), the cell-free wound surface was measured between the wound edges, averaged between the fields of view and triplicates, and the percentage of wound closure was calculated according to the following equation: $\frac{\left(A0-At\right)}{A0}\times 100$.

*A*0 is the initial wound area and *At* is the wound area *n* hours after the initial scratch, both in μm^2^.

### 4.5. Western Blot

Western blot analysis was performed as previously described [71]. Briefly, podocyte cells were counted using a hemocytometer and plated in 6-well plates at a density of 25 × 10^4^/well. The plates were incubated overnight under growth conditions to allow for cell recovery and exponential growth. After overnight incubation, the cells were serum-starved in 0.1% FBS RPMI for 24 h. Following overnight starvation, the cells were treated with 1 µg/mL of COE for 10, 30, 60, and 120 min, respectively.

In other experiments, podocytes cells were treated with 1mM sodium orthovanadate for 24 h in the presence or absence of COE (2 µg/mL).

A total of 10^6^ cells for each treatment were pelleted and lysed in RIPA lysis buffer supplemented with protease inhibitor cocktail (Roche, Basel, Switzerland). Cell lysates were centrifuged at 12,000× *g* at 4 °C for 15 min, and protein concentration was measured using the Bradford assay kit (Thermo Fisher, Waltham, MA, USA). Proteins were separated by SDS–PAGE in reducing conditions and transferred to nitrocellulose membrane. After blocking with 5% milk, the membrane was incubated overnight at 4 °C with the appropriate primary antibody: anti-phospho-AKT1 (S473), anti-phospho-p38 (phospho T180 + Y182), total anti-(AKT1 + AKT2 + AKT3) antibody and total anti-p38 alpha/beta MAPK, anti-PARP, anti-caspase-3, and anti-catalse antibodies (all antibodies were purchased from Abcam, Cambridge, UK). After overnight incubation, the membranes were washed, then incubated with HRP-conjugated secondary antibody for 1 h at room temperature and revealed using the ECL substrate using the Chemidoc^TM^ MP Imaging System (Biorad, Hercules, CA, USA). Monoclonal anti-beta actin antibody was used for protein loading control. Densitometric analysis was performed using ImageJ.

### 4.6. In Vivo Model and Experimental Design

#### 4.6.1. Study Protocol

Forty Sprague Dawley male rats, aged 8 weeks, were obtained from the animal facility at the Lebanese American University and were housed under controlled conditions. Food and water were provided ad libitum. All efforts were made to minimize animal suffering and reduce the number of animals used. Rats were randomly divided into five groups: Group 1 (*n* = 8) was injected with 0.2 mL of intraperitoneal vehicle solution consisting of ethanol–Tween 80–PBS (1:1:18) daily for 10 days. Group 2 (*n* = 8) was injected with 10 mg/kg intraperitoneal sodium orthovanadate daily for 10 days. Group 3 (*n* = 8) was injected with 10 mg/kg intraperitoneal sodium orthovanadate daily for 10 days, and 5 mg/kg intraperitoneal COE from day 4 to day 10. Group 4 (*n* = 8) was injected with 10 mg/kg intraperitoneal sodium orthovanadate daily for 10 days and 10 mg/kg intraperitoneal COE from day 4 to day 10. Group 5 (*n* = 8) was injected with 10 mg/kg intraperitoneal sodium orthovanadate daily for 10 days and 20 mg/kg intraperitoneal COE from day 4 to day 10. All animals were weighed on days 1, 3, 5, 7, 9, and 11. Rats were sacrificed on day 11 of the experiment.

#### 4.6.2. Serum Analysis

Blood samples were collected immediately after the sacrifice of rats. EDTA was added to prevent clotting of the samples in a ratio of 25 μL (0.5 M) to each 1 mL of blood collected. Then, the collected blood samples were centrifuged at 3000 rpm for 20 min at 4 °C for efficient separation and the recovery of plasma. The concentrations of renal function biological parameters, including serum creatinine, urea, and electrolytes, were analyzed.

#### 4.6.3. Histopathologic Testing

Organ pathology was performed as previously described [72]. Briefly, the kidneys and hearts of all groups were dissected out and weighed. The organs were fixed in 10% neutral buffered formalin. Then, they were taken, dehydrated, and embedded in paraffin. Tissue sections from the paraffin-embedded blocks were mounted on glass slides, deparaffinized, rehydrated, stained with Hematoxylin and Eosin, and then examined microscopically.

### 4.7. Statistical Analysis

Data analyses are expressed as mean ± standard error of mean (SEM). For two-group comparisons, unpaired t-tests were used. Differences between multiple groups were evaluated using one-way ANOVA followed by Bonferroni’s post hoc test for multiple comparisons. The significance level was accepted with *p* values <0.05 (*), <0.01 (**), <0.001 (***), and <0.0001 (****). Statistical analysis was performed using GraphPad Prism 8.4.

## Figures and Tables

**Table 1:** Body, absolute, and relative organ weights of control rats and rats treated with sodium orthovanadate and COE.

	Control (Vehicle)	Vanadate	Vanadate + COE (5 mg/kg)	Vanadate + COE (10 mg/kg)	Vanadate + COE (20 mg/kg)
Body Weight Gain (g)	85.50 ± 1.73	58.38 ± 5.32 ***	52.57 ± 4.36 ****	51.88 ± 3.72 ****	51.43 ± 5.47 ****
Absolute Kidney Wt (g)	0.81 ± 0.03	0.80 ± 0.02	0.72 ± 0.03	0.82 ± 0.03	0.72 ± 0.02
Relative Kidney Wt (g)	0.42 ± 0.01	0.45 ± 0.02	0.45 ± 0.01	0.47 ± 0.01 *	0.45 ± 0.01
Absolute Heart Wt (g)	0.74 ± 0.02	0.64 ± 0.03 **	0.58 ± 0.03	0.66 ± 0.03 **	0.59 ± 0.04
Relative Heart Wt (g)	0.39 ± 0.01	0.36 ± 0.01	0.37 ± 0.01	0.38 ± 0.01	0.37 ± 0.03

Values are means ± SEM of eight rats in each group. * *p* value < 0.05; ** *p* < 0.01; *** *p* < 0.001; **** *p* < 0.0001 vs. control group.

**Table 2:** Serum electrolyte levels of control rats and rats treated with sodium orthovanadate and COE.

	Control (Vehicle)	Vanadate	Vanadate + COE (5 mg/kg)	Vanadate + COE (10 mg/kg)	Vanadate + COE (20 mg/kg)
Na^+^ (mmol/L)	135.63 ± 1.21	137 ± 1.21	138.86 ± 0.67	138.75 ± 1.63	139 ± 1.59
K^+^ (mmol/L)	5.19 ± 0.45	5.29 ± 0.34	5.68 ± 0.47	6.59 ± 0.56	6.28 ± 0.72
Cl^−^ (mmol/L)	94.50 ± 0.65	95.89 ± 0.95	95.86 ± 1.32	95.56 ± 0.49	97.90 ± 0.55

Values are means ± SEM of eight rats in each group.

## Data Availability

The data presented in this study are available on request from the corresponding author.

## References

[1] López-Novoa J.M., Martínez-Salgado C., Rodríguez-Peña A.B., López-Hernández F.J. (2010). Common pathophysiological mechanisms of chronic kidney disease: Therapeutic perspectives. Pharmacol. Ther..

[2] Aoun M., Makkouk J., Ammar W. (2019). Ultrapure water in haemodialysis: A step towards better quality in Lebanon. East. Mediterr. Health J..

[3] Goyal A., Daneshpajouhnejad P., Hashmi M.F., Bashir K. (2024). Acute Kidney Injury. StatPearls.

[4] Rehder D. (2012). The potentiality of vanadium in medicinal applications. Future Med. Chem..

[5] Irving E., Stoker A.W. (2017). Vanadium Compounds as PTP Inhibitors. Molecules.

[6] Higashi Y., Bello-Reuss E. (1980). Effects of sodium orthovanadate on whole kidney and single nephron function. Kidney Int..

[7] Boscolo P., Carmignani M., Volpe A.R., Felaco M., Del Rosso G., Porcelli G., Giuliano G. (1994). Renal toxicity and arterial hypertension in rats chronically exposed to vanadate. Occup. Environ. Med..

[8] Soussi A., Abdennabi R., Ghorbel F., Murat J.C., El Feki A.F. (2017). Ameliorated Effects of (-)-Epigallocatechin Gallate Against Toxicity Induced by Vanadium in the Kidneys of Wistar Rats. Biol. Trace Elem. Res..

[9] Yamada K.M., Araki M. (2001). Tumor suppressor PTEN: Modulator of cell signaling, growth, migration and apoptosis. J. Cell Sci..

[10] Leslie N.R., Downes C.P. (2002). PTEN: The down side of PI 3-kinase signalling. Cell Signal.

[11] Bu L., Wang H., Pan J.-a., Chen L., Xing F., Wu J., Li S., Guo D. (2021). PTEN suppresses tumorigenesis by directly dephosphorylating Akt. Signal Transduct. Target. Ther..

[12] Lan R., Geng H., Polichnowski A.J., Singha P.K., Saikumar P., McEwen D.G., Griffin K.A., Koesters R., Weinberg J.M., Bidani A.K. (2012). PTEN loss defines a TGF-β-induced tubule phenotype of failed differentiation and JNK signaling during renal fibrosis. Am. J. Physiol. Renal Physiol..

[13] Chen J.K., Nagai K., Chen J., Plieth D., Hino M., Xu J., Sha F., Ikizler T.A., Quarles C.C., Threadgill D.W. (2015). Phosphatidylinositol 3-kinase signaling determines kidney size. J. Clin. Investig..

[14] Lin J., Shi Y., Peng H., Shen X., Thomas S., Wang Y., Truong L.D., Dryer S.E., Hu Z., Xu J. (2015). Loss of PTEN promotes podocyte cytoskeletal rearrangement, aggravating diabetic nephropathy. J. Pathol..

[15] Huang C.-Y., Tan T.-H. (2012). DUSPs, to MAP kinases and beyond. Cell Biosci..

[16] Owens D.M., Keyse S.M. (2007). Differential regulation of MAP kinase signalling by dual-specificity protein phosphatases. Oncogene.

[17] Schiffer M., Bitzer M., Roberts I.S., Kopp J.B., ten Dijke P., Mundel P., Böttinger E.P. (2001). Apoptosis in podocytes induced by TGF-beta and Smad7. J. Clin. Investig..

[18] Wharram B.L., Goyal M., Wiggins J.E., Sanden S.K., Hussain S., Filipiak W.E., Saunders T.L., Dysko R.C., Kohno K., Holzman L.B. (2005). Podocyte depletion causes glomerulosclerosis: Diphtheria toxin-induced podocyte depletion in rats expressing human diphtheria toxin receptor transgene. J. Am. Soc. Nephrol..

[19] An N., Bassil K., Al Jowf G.I., Steinbusch H.W.M., Rothermel M., de Nijs L., Rutten B.P.F. (2021). Dual-specificity phosphatases in mental and neurological disorders. Prog. Neurobiol..

[20] Li H., Xiong J., Du Y., Huang Y., Zhao J. (2022). Dual-Specificity Phosphatases and Kidney Diseases. Kidney Dis..

[21] Abdallah M.S., Kennedy C.R.J., Stephan J.S., Khalil P.A., Mroueh M., Eid A.A., Faour W.H. (2018). Transforming growth factor-β1 and phosphatases modulate COX-2 protein expression and TAU phosphorylation in cultured immortalized podocytes. Inflamm. Res..

[22] Hartsel J.A., Eades J., Hickory B., Makriyannis A., Gupta R.C. (2016). Chapter 53—*Cannabis sativa* and Hemp. Nutraceuticals.

[23] Voeks R. (2014). Cannabis: Evolution and Ethnobotany. AAG Rev. Books.

[24] Lozano I. (1997). Therapeutic use of *Cannibis sativa* L. in Arab medicine. Asclepio.

[25] Shebaby W., Saliba J., Faour W.H., Ismail J., El Hage M., Daher C.F., Taleb R.I., Nehmeh B., Dagher C., Chrabieh E. (2021). In vivo and in vitro anti-inflammatory activity evaluation of Lebanese *Cannabis sativa* L. ssp. indica (Lam.). J. Ethnopharmacol..

[26] Bylan D., Khalil A., Shebaby W., Habchy C., Nasser S., Faour W.H., Mroueh M. (2024). Lebanese cannabis oil extract protected against folic acid-induced kidney fibrosis in rats. PLoS ONE.

[27] Khalil A., Al Toufaily S., Shebaby W., Hage M.E., Mroue D., Faour W., Mroueh M. (2025). Lebanese *Cannabis sativa* L. extract protects from cisplatin-induced nephrotoxicity in mice by inhibiting podocytes apoptosis. J. Cannabis Res..

[28] Andre C.M., Hausman J.F., Guerriero G. (2016). *Cannabis sativa*: The Plant of the Thousand and One Molecules. Front. Plant Sci..

[29] Campos A.C., Fogaça M.V., Sonego A.B., Guimarães F.S. (2016). Cannabidiol, neuroprotection and neuropsychiatric disorders. Pharmacol. Res..

[30] Sangiovanni E., Fumagalli M., Pacchetti B., Piazza S., Magnavacca A., Khalilpour S., Melzi G., Martinelli G., Dell’Agli M. (2019). *Cannabis sativa* L. extract and cannabidiol inhibit in vitro mediators of skin inflammation and wound injury. Phytother. Res..

[31] Koppel B.S., Brust J.C., Fife T., Bronstein J., Youssof S., Gronseth G., Gloss D. (2014). Systematic review: Efficacy and safety of medical marijuana in selected neurologic disorders: Report of the Guideline Development Subcommittee of the American Academy of Neurology. Neurology.

[32] Feizi A., Jafari M.R., Hamedivafa F., Tabrizian P., Djahanguiri B. (2008). The preventive effect of cannabinoids on reperfusion-induced ischemia of mouse kidney. Exp. Toxicol. Pathol..

[33] Fouad A.A., Al-Mulhim A.S., Jresat I. (2012). Cannabidiol treatment ameliorates ischemia/reperfusion renal injury in rats. Life Sci..

[34] Ucibior A., Gołębiowska D., Adamczyk A., Niedźwiecka I., Fornal E. (2014). The renal effects of vanadate exposure: Potential biomarkers and oxidative stress as a mechanism of functional renal disorders--preliminary studies. BioMed Res. Int..

[35] Tejada T., Catanuto P., Ijaz A., Santos J.V., Xia X., Sanchez P., Sanabria N., Lenz O., Elliot S.J., Fornoni A. (2008). Failure to phosphorylate AKT in podocytes from mice with early diabetic nephropathy promotes cell death. Kidney Int..

[36] Cai C., Hu W., Zhang Y., Hu X., Yang S., Qiu H., Wang R., Ma M., Qiu Y., Chu T. (2021). BCI Suppresses RANKL-Mediated Osteoclastogenesis and Alleviates Ovariectomy-Induced Bone Loss. Front. Pharmacol..

[37] Thompson E.M., Patel V., Rajeeve V., Cutillas P.R., Stoker A.W. (2022). The cytotoxic action of BCI is not dependent on its stated DUSP1 or DUSP6 targets in neuroblastoma cells. FEBS Open Bio.

[38] Silva-Nolasco A.M., Camacho L., Saavedra-Díaz R.O., Hernández-Abreu O., León I.E., Sánchez-Lombardo I. (2020). Kinetic Studies of Sodium and Metforminium Decavanadates Decomposition and In Vitro Cytotoxicity and Insulin- Like Activity. Inorganics.

[39] Minaei Beyrami S., Khadem Ansari M.H., Rasemi Y., Shakib N., Karimi P. (2018). Complete inhibition of phosphatase and tensin homolog promotes the normal and oxygen-glucose deprivation/reperfusion-injured PC12 cells to cell death. J. Cardiovasc. Thorac. Res..

[40] Zhou J., Jia L., Hu Z., Wang Y. (2017). Pharmacological Inhibition of PTEN Aggravates Acute Kidney Injury. Sci. Rep..

[41] Kim J., Choi J.Y., Seo J., Choi I.S. (2021). Neuroprotective Effect of Cannabidiol Against Hydrogen Peroxide in Hippocampal Neuron Culture. Cannabis Cannabinoid Res..

[42] Fouda M.A., Ghovanloo M.R., Ruben P.C. (2020). Cannabidiol protects against high glucose-induced oxidative stress and cytotoxicity in cardiac voltage-gated sodium channels. Br. J. Pharmacol..

[43] Li Y., Hao D., Wei D., Xiao Y., Liu L., Li X., Wang L., Gan Y., Yan W., Ke B. (2022). Photoprotective Effects of Cannabidiol against Ultraviolet-B-Induced DNA Damage and Autophagy in Human Keratinocyte Cells and Mouse Skin Tissue. Molecules.

[44] Robitaille A.C., Caron E., Zucchini N., Mukawera E., Adam D., Mariani M.K., Gélinas A., Fortin A., Brochiero E., Grandvaux N. (2017). DUSP1 regulates apoptosis and cell migration, but not the JIP1-protected cytokine response, during Respiratory Syncytial Virus and Sendai Virus infection. Sci. Rep..

[45] Moncho-Amor V., Ibañez de Cáceres I., Bandres E., Martínez-Poveda B., Orgaz J.L., Sánchez-Pérez I., Zazo S., Rovira A., Albanell J., Jiménez B. (2011). DUSP1/MKP1 promotes angiogenesis, invasion and metastasis in non-small-cell lung cancer. Oncogene.

[46] Ahmad M.K., Abdollah N.A., Shafie N.H., Yusof N.M., Razak S.R.A. (2018). Dual-specificity phosphatase 6 (DUSP6): A review of its molecular characteristics and clinical relevance in cancer. Cancer Biol. Med..

[47] Wu Q.N., Liao Y.F., Lu Y.X., Wang Y., Lu J.H., Zeng Z.L., Huang Q.T., Sheng H., Yun J.P., Xie D. (2018). Pharmacological inhibition of DUSP6 suppresses gastric cancer growth and metastasis and overcomes cisplatin resistance. Cancer Lett..

[48] Gao X., Wang N., Wu S., Cui H., An X., Yang Y. (2019). Long non-coding RNA FER1L4 inhibits cell proliferation and metastasis through regulation of the PI3K/AKT signaling pathway in lung cancer cells. Mol. Med. Rep..

[49] Yue C., Bai Y., Piao Y., Liu H. (2021). DOK7 Inhibits Cell Proliferation, Migration, and Invasion of Breast Cancer via the PI3K/PTEN/AKT Pathway. J. Oncol..

[50] Denhez B., Rousseau M., Dancosst D.A., Lizotte F., Guay A., Auger-Messier M., Côté A.M., Geraldes P. (2019). Diabetes-Induced DUSP4 Reduction Promotes Podocyte Dysfunction and Progression of Diabetic Nephropathy. Diabetes.

[51] Reiser J., Pixley F.J., Hug A., Kriz W., Smoyer W.E., Stanley E.R., Mundel P. (2000). Regulation of mouse podocyte process dynamics by protein tyrosine phosphatases rapid communication. Kidney Int..

[52] Anis O., Vinayaka A.C., Shalev N., Namdar D., Nadarajan S., Anil S.M., Cohen O., Belausov E., Ramon J., Mayzlish Gati E. (2021). Cannabis-Derived Compounds Cannabichromene and Δ9-Tetrahydrocannabinol Interact and Exhibit Cytotoxic Activity against Urothelial Cell Carcinoma Correlated with Inhibition of Cell Migration and Cytoskeleton Organization. Molecules.

[53] Nabissi M., Morelli M.B., Offidani M., Amantini C., Gentili S., Soriani A., Cardinali C., Leoni P., Santoni G. (2016). Cannabinoids synergize with carfilzomib, reducing multiple myeloma cells viability and migration. Oncotarget.

[54] Gerasymchuk M., Robinson G.I., Groves A., Haselhorst L., Nandakumar S., Stahl C., Kovalchuk O., Kovalchuk I. (2022). Phytocannabinoids Stimulate Rejuvenation and Prevent Cellular Senescence in Human Dermal Fibroblasts. Cells.

[55] Luo H., Rossi E., Saubamea B., Chasseigneaux S., Cochois V., Choublier N., Smirnova M., Glacial F., Perrière N., Bourdoulous S. (2019). Cannabidiol Increases Proliferation, Migration, Tubulogenesis, and Integrity of Human Brain Endothelial Cells through TRPV2 Activation. Mol. Pharm..

[56] Giacoppo S., Pollastro F., Grassi G., Bramanti P., Mazzon E. (2017). Target regulation of PI3K/Akt/mTOR pathway by cannabidiol in treatment of experimental multiple sclerosis. Fitoterapia.

[57] Ivanov V.N., Grabham P.W., Wu C.C., Hei T.K. (2021). Author Correction: Inhibition of autophagic flux differently modulates cannabidiol-induced death in 2D and 3D glioblastoma cell cultures. Sci. Rep..

[58] de la Torre A., Granero S., Mayayo E., Corbella J., Domingo J.L. (1999). Effect of age on vanadium nephrotoxicity in rats. Toxicol. Lett..

[59] Wilk A., Szypulska-Koziarska D., Wiszniewska B. (2017). The toxicity of vanadium on gastrointestinal, urinary and reproductive system, and its influence on fertility and fetuses malformations. Postepy Hig. Med. Dosw..

[60] Eiam-Ong S., Nakchui Y., Chaipipat M., Eiam-Ong S. (2018). Vanadate-Induced Renal cAMP and Malondialdehyde Accumulation Suppresses Alpha 1 Sodium Potassium Adenosine Triphosphatase Protein Levels. Toxicol. Res..

[61] Zendeboodi S., Esmaili A., Movahed A., Fatemikia H., Jamshidi A., Nazari M., Heydari H., Seyedian R. (2019). The attenuative effects of oral resveratrol on renalchanges induced by vanadium injection in rats. J. Ren. Inj. Prev..

[62] Al-Bayati M.A., Xie Y., Mohr F.C., Margolin S.B., Giri S.N. (2002). Effect of pirfenidone against vanadate-induced kidney fibrosis in rats. Biochem. Pharmacol..

[63] Marouane W., Soussi A., Murat J.C., Bezzine S., El Feki A. (2011). The protective effect of Malva sylvestris on rat kidney damaged by vanadium. Lipids Health Dis..

[64] Pertwee R.G. (2008). The diverse CB1 and CB2 receptor pharmacology of three plant cannabinoids: Delta9-tetrahydrocannabinol, cannabidiol and delta9-tetrahydrocannabivarin. Br. J. Pharmacol..

[65] Levinsohn E.A., Hill K.P. (2020). Clinical uses of cannabis and cannabinoids in the United States. J. Neurol. Sci..

[66] Mukhopadhyay P., Baggelaar M., Erdelyi K., Cao Z., Cinar R., Fezza F., Ignatowska-Janlowska B., Wilkerson J., van Gils N., Hansen T. (2016). The novel, orally available and peripherally restricted selective cannabinoid CB2 receptor agonist LEI-101 prevents cisplatin-induced nephrotoxicity. Br. J. Pharmacol..

[67] Jourdan T., Szanda G., Rosenberg A.Z., Tam J., Earley B.J., Godlewski G., Cinar R., Liu Z., Liu J., Ju C. (2014). Overactive cannabinoid 1 receptor in podocytes drives type 2 diabetic nephropathy. Proc. Natl. Acad. Sci. USA.

[68] Jourdan T., Park J.K., Varga Z.V., Pálóczi J., Coffey N.J., Rosenberg A.Z., Godlewski G., Cinar R., Mackie K., Pacher P. (2018). Cannabinoid-1 receptor deletion in podocytes mitigates both glomerular and tubular dysfunction in a mouse model of diabetic nephropathy. Diabetes Obes. Metab..

[69] Barutta F., Bellini S., Mastrocola R., Gambino R., Piscitelli F., di Marzo V., Corbetta B., Vemuri V.K., Makriyannis A., Annaratone L. (2018). Reversal of albuminuria by combined AM6545 and perindopril therapy in experimental diabetic nephropathy. Br. J. Pharmacol..

[70] Mukhopadhyay P., Rajesh M., Pan H., Patel V., Mukhopadhyay B., Bátkai S., Gao B., Haskó G., Pacher P. (2010). Cannabinoid-2 receptor limits inflammation, oxidative/nitrosative stress, and cell death in nephropathy. Free Radic. Biol. Med..

[71] El Zein N., Abdallah M.S., Daher C.F., Mroueh M., Stephan J., Bahous S.A., Eid A., Faour W.H. (2019). Ghrelin modulates intracellular signalling pathways that are critical for podocyte survival. Cell Biochem. Funct..

[72] Dagher-Hamalian C., Stephan J., Zeeni N., Harhous Z., Shebaby W.N., Abdallah M.S., Faour W.H. (2020). Ghrelin-induced multi-organ damage in mice fed obesogenic diet. Inflamm. Res..

